# Alterations of structural–functional connectivity coupling in older adults with depressive symptoms

**DOI:** 10.1017/S0033291725101657

**Published:** 2025-10-03

**Authors:** Ting Li, Haishuo Xia, Shaokun Zhao, Jiawen Liu, Biying Peng, Ziyun Li, Birong Ge, Xin Li, Zhanjun Zhang

**Affiliations:** 1 State Key Laboratory of Cognitive Neuroscience and Learning, Beijing Normal University, Beijing, China; 2 BABRI Centre, Beijing Normal University, Beijing, China; 3 Department of Radiology, 7T Magnetic Resonance Translational Medicine Research Center, Southwest Hospital, Army Medical University (Third Military Medical University), Chongqing, China; 4 Beijing Huilongguan Hospital, Peking University Huilongguan Clinical Medical School, Beijing, China; 5 Integrating Innovative Institute of Chinese and Western Medicine, Shandong First Medical University & Shandong Academy of Medical Sciences, Jinan, Shandong, China

**Keywords:** Depressive symptoms, older adults, structural-functional connectivity coupling

## Abstract

**Background:**

Prior research indicates that both structural and functional networks are compromised in older adults experiencing depressive symptoms. However, the potential impact of abnormal interactions between brain structure and function remains unclear. This study investigates alterations in structural–functional connectivity coupling (SFC) among older adults with depressive symptoms, and explores how these changes differ depending on the presence of physiological comorbidities.

**Methods:**

We used multimodal neuroimaging data (dMRI/rs-fMRI) from 415 older adults with depressive symptoms and 415 age-matched normal controls. Subgroups were established within the depressive group based on the presence of hypertension, hyperlipidemia, diabetes, cerebrovascular disease, and sleep disorders. We examined group and subgroup differences in SFC and tracked its alterations in relation to symptom progression.

**Results:**

Older adults with depressive symptoms showed significantly increased SFC in the ventral attention network compared with normal controls. Moreover, changes in SFC within the subcortical network, especially in the left amygdala, were closely linked to symptom progression. Subgroup analyses further revealed heterogeneity in SFC changes, with certain physiological health factors, such as metabolic diseases and sleep disorders, contributing to distinct neural mechanisms underlying depressive symptoms in this population.

**Conclusions:**

This study identifies alterations in SFC related to depressive symptoms in older adults, primarily within the ventral attention and subcortical networks. Subgroup analyses highlight the heterogeneous SFC changes associated with metabolic diseases and sleep disorders. These findings highlight SFC may serve as potential markers for more personalized interventions, ultimately improving the clinical management of depression in older adults.

## Introduction

Depressive symptoms and major depressive disorder are prevalent among older adults, influenced by factors like chronic medical conditions, sleep quality, and significant life events (Hu et al., 2022; Zenebe, Akele, W/Selassie, & Necho, 2021). Even subthreshold depressive symptoms can signal poor health outcomes and increased mortality risk (Agustini et al., 2022). However, depression in older adults is often underdiagnosed and undertreated, as symptoms are often mistaken for normal aging, responses to stress, or other physical ailments (Agustini et al., 2022; Saczynski et al., 2010; Sadavoy, 2009; Wilkinson, Ruane, & Tempest, 2018; Wu, Schimmele, & Chappell, 2012). Therefore, advancing our understanding of the neurobiological basis of depressive symptoms in older adults and how these mechanisms interact with common physical comorbidities is crucial to enable timely screening and effective interventions.

Recent research indicates that the neurobiological basis of depressive symptoms in older adults involves alterations across multiple brain modalities. Functional magnetic resonance imaging (fMRI) studies revealed changes in both reduced integration in key large-scale networks, such as the default mode network (DMN), central control network, and frontolimbic network (Alexopoulos et al., 2012; Csukly et al., 2024; Jiang et al., 2025; Touron et al., 2022); and abnormal local connectivity in specific regions like the anterior cingulate cortex, hippocampus, and amygdala (Leal, Noche, Murray, & Yassa, 2017; Li, Ma, Yu, He, & Li, 2014; Liu et al., 2024; Touron et al., 2023). In addition, structural changes include decreased grey matter volume in the hippocampus, increased white matter hyperintensity volume, and decreased white matter integrity in the fornix and the posterior parts of the cingulum and corpus callosum (Bogoian et al., 2024; Csukly et al., 2024; Touron et al., 2022). While there is clear evidence for both structural and functional brain alterations, the complex interactions between these changes during the progression of depressive symptoms in older adults remain unclear.

A promising framework for understanding this interaction is structural–functional connectivity coupling (SFC). SFC quantifies the alignment between the brain’s anatomical pathways and synchronized functional activity (Fotiadis et al., 2024). Across psychiatric disorders, SFC disturbances are common (Collin, Scholtens, Kahn, Hillegers, & van den Heuvel, 2017; Skudlarski et al., 2010), yet depression appears to manifest a distinct, network- and age-specific pattern. During adolescence, patients with depression show increased coupling in the DMN and Visual Network (VN), indicating a tight anchoring of functional communications to anatomical pathways (Xu et al., 2024). In young adults, DMN coupling remains increased whereas coupling in the frontoparietal network (FPN) decreases, implying regional specificity and hierarchical nature of SFC abnormalities (Chu et al., 2024; Jiang et al., 2019). In late life, among older women, SFC reduced globally as symptom severity increases (Suo, Chen, Kemp, Wu, & Wang, 2025). Collectively, these findings suggest SFC abnormalities may contribute to the pathophysiology of depression. SFC may serves as sensitive indicators of network-level dysfunction in depression, revealing context-dependent alterations that single-modality approaches may overlook.

Critically, depressive symptoms in older adults are heterogeneous largely because physiological factors like chronic diseases and sleep disorders (Baglioni et al., 2016; Valkanova & Ebmeier, 2013). Vascular and metabolic comorbidities induce distinctive neural patterns: hypertension accelerates grey matter atrophy in cingulate regions (Meurs et al., 2015), dyslipidemia reduces neurite density in the corpus callosum and internal capsule (Mahemuti, 2025), and diabetes triggers neuroinflammatory and neuroendocrine cascades that reshape serotonin pathways and wider circuits (Fanelli et al., 2025; Khawagi, 2024). Cerebrovascular disease further fragments white-matter tracts and disrupts default-mode coupling, aligning with vascular-depression theory (Herrmann, Le Masurier, & Ebmeier, 2008; Schulz, Malherbe, Cheng, Thomalla, & Schlemm, 2021; Wu, 2024). Independently, sleep disorders compound these effects by eroding reward-related subcortical volumes, perturbing limbic–prefrontal and salience networks, and aggravating microvascular damage (Bagherzadeh-Azbari et al., 2019; de Lange et al., 2025; Kerner & Roose, 2016; Peng et al., 2025). These findings underscore the importance to consider these subgroups to reveal specific neural substrates underlying older adults with depressive symptoms.

Therefore, the primary goal of this study is to examine SFC alterations in older adults with depressive symptoms by integrating diffusion MRI and resting-state fMRI data. The specific objectives are to: (1) identify global alterations in SFC associated with depressive symptoms by comparing SFC patterns between older adults with and without depressive symptoms; (2) explore the neuropathological heterogeneity of depression by examining SFC patterns across different subgroups within the depressive group; and (3) capture the trajectory of SFC over the course of depressive symptoms, aiming to identify sensitive neurobiological marker that reflect symptom progression by examining the relationship between SFC and the severity of depressive symptoms.

## Methods and materials

### Participants

We recruited 415 older adults with depressive symptoms (DS) and 415 age-matched normal controls (NC) (Yang et al., 2021). Depressive symptoms were assessed by the self-reported Geriatric Depression Scale (GDS, Yesavage et al., 1982) (Supplementary Material). Detailed sociodemographic and clinical characteristics of all participants are presented in Table 1. Participants were eligible for inclusion if they met the following criteria: (1) age ≥50 years; (2) normal cognitive function and intact activities of daily living, as assessed by clinical interview and standardized scales; (3) a minimum of 6 years of formal education; (4) no history of neurological disorders (such as stroke, Parkinson’s disease, or epilepsy), psychiatric diseases (other than depression), or other brain-related illnesses; (5) no significant head injury; (6) absence of major systemic diseases (such as malignancy or severe cardiac, hepatic, or renal dysfunction); and (7) availability of complete clinical assessments, Geriatric Depression Scale (GDS) scores, and high-quality MRI data. Exclusion criteria included: (1) presence of severe sensory deficits (e.g. blindness, deafness) interfering with assessment; (2) MRI contraindications (e.g. metallic implants, claustrophobia); (3) current or recent use (within 3 months) of medications affecting central nervous system function; (4) significant structural abnormalities detected on MRI (e.g. large infarcts, tumors, hydrocephalus); or (5) inability to provide informed consent. The institutional review board of Beijing Normal University approved the study, and each participant provided written informed consent.

**Table 1.** Demographic information of participants

Characteristic	Normal control (*n* = 415)	Depressive symptom (*n* = 415)	*t/χ* ^2^	*p*
Demographic information				
Age (years)	65.8 ± 6.00	66 ± 6.64	−0.455	0.649
Gender (M/F)	140/275	146/269	0.133	0.715
Education (years)	11.07 ± 2.86	11.03 ± 2.9	0.211	0.833
Marriage (married/unmarried)	355/48	338/62	1.895	0.169
Clinical characteristics				
Hypertension	168 (40.48%)	210 (50.72%)	10.784	0.005
Hyperlipemia	160 (38.55%)	185 (44.69%)	3.28	0.194
Diabetes	126 (30.36%)	117 (28.26%)	0.681	0.711
Cerebral vascular disease	66 (15.9%)	122 (29.47%)	23.037	<0.001

We further divided participants in both the DS and NC groups into subgroups based on the presence of chronic diseases (including hypertension, hyperlipemia, diabetes, and cerebral vascular disease) and sleep disorders. The status of each disease was determined by self-reported physician’s diagnoses, while sleep disorder status was assessed using the Pittsburgh Sleep Quality Index (PSQI), with a total score >5 indicating a sleep disorder (Buysse, Reynolds, Monk, Berman, & Kupfer, 1989). Specifically, for each condition, the subgroups were grouped as follows: (1) participants in the NC group without the condition, (2) participants in the DS group without the condition, and (3) participants in the DS group with the condition. Detailed sociodemographic and clinical characteristics of all subgroup participants are shown in Supplementary Table S3.

### Image acquisition and preprocessing

T1-weighted images, resting-state fMRI, and diffusion tensor imaging (DTI) were acquired by scanning on a 3.0 T SIEMENS PRISMA scanner at the Imaging Centre for Brain Research at Beijing Normal University. Image acquisition parameters are provided in Supplemental Information. Resting-state fMRI time series and DTI imaging were processed with fMRIPrep (Esteban et al., 2019) and QSIPrep (Cieslak et al., 2021), respectively. The preprocessing of T1-weighted images included bias correction, skull-stripping, and normalizing to MNI (Montreal Neurological Institute) standard space. The preprocessing of resting-state fMRI included motion correction, slice-timing correction, susceptibility distortion correction, and registration. For DTI data, including denoising, B1-field correction, eddy current correction, DTI-T1-weighted alignment, and model fitting.

### Construction of the structural connectivity

The construction of the White Matter Fiber Number Network based on the Brainnetome Atlas (region definition in Supplementary Table S1) (Fan et al., 2016) comprises 246 regions of interest (ROIs). Probabilistic fibre tracking was conducted in individual spaces using the MRtrix3 fibre tracking algorithm (Tournier et al., 2019) to generate probabilistic fibre bundles. Tracking was performed along the most probable fibre directions between each ROI, ensuring that the algorithm considered fibre orientation and path continuity to enhance the accuracy and reliability of the tracking results. The resulting probabilistic fibre bundles underwent filtering and post-processing. The filtering step involved eliminating fibre bundles that did not meet specific criteria, such as being excessively short or having inconsistent orientations, to ensure the quality and reliability of the final output. Finally, streamline counts were normalized by the mean volumes of the seed and target region, resulting in a normalized connection weight matrix.

### Construction of the functional connectomes

fMRI frames with framewise displacement >0.5 mm or standardized DVARS values exceeding 1.5 (representing the spatial standard deviation of successive difference images) were excluded from the analysis. The remaining preprocessed BOLD (blood oxygenation level-dependent) data were then linearly detrended, band-pass filtered between 0.01 and 0.08 Hz, and regressed for 36 confounding variables (Supplementary Material). The nilearn.image.clean_image function was used to standardize the cleaned data. After preprocessing and denoising, functional connectivity between each pair of brain regions was calculated as the Fisher *z*-transformed Pearson correlation coefficient between the mean regional residual BOLD time series, resulting in a 246-by-246 weighted adjacent matrix for each participant.

### Calculation of SFC coupling

Regional connectivity profiles were derived from each column of a participant’s structural or functional connectivity matrix, represented as vectors indicating the connectivity strength from a single network node to all other nodes in the network. Structure–function coupling was then assessed by calculating the Spearman rank correlation between nonzero elements of regional structural and functional connectivity profiles (Collin et al., 2017).

### Statistical analysis

**Figure 1.**
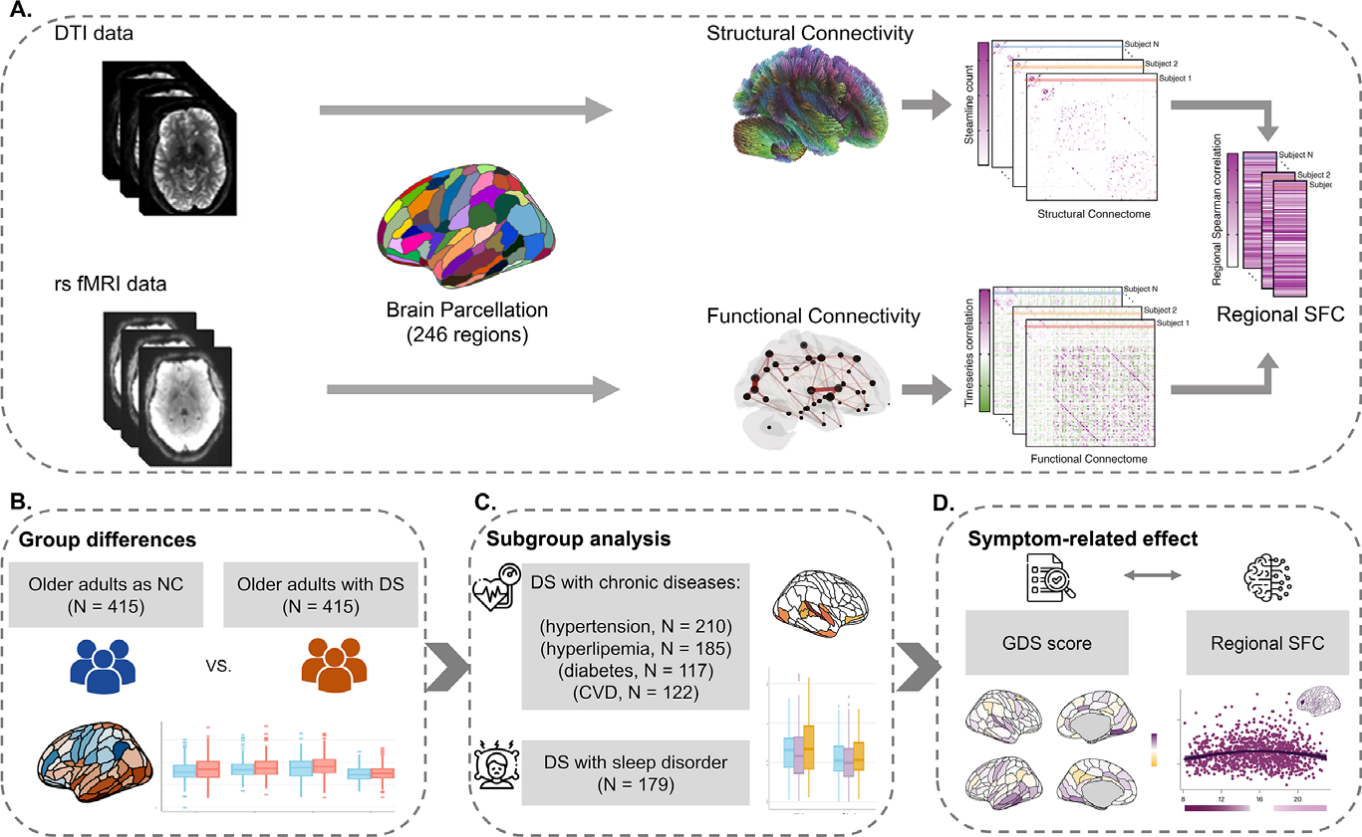
Study design and data analysis pipeline. (a) The pipeline for calculating the SFC of each brain region. First, functional and structural connectivity with 246 regions was calculated for each participant. Second, the SFC for each brain region was assessed by calculating the Spearman rank correlation between nonzero elements of regional structural and functional connectivity profiles. (b) Group differences analysis in regional SFC between DS and NC older adults. (c) Subgroup analysis of the SFC variations between DS subgroup with different physiological health factors and normal controls. (d) Association analysis between depressive symptom progression and regional SFC. *Note*: DTI, ‘diffusion tensor imaging’; rs fMRI, ‘resting-state functional magnetic resonance imaging’; SFC, ‘structural-functional connectivity coupling’; DS, ‘depressive symptoms’; NC, ‘normal controls’; GDS, ‘geriatric depression scale’.

The study design and data analysis pipeline are presented in Figure 1. Statistical analyses were performed using R, version 4.2.2 (R Project for Statistical Computing). Multiple comparisons were controlled using the false discovery rate (FDR; Benjamini–Hochberg method) applied globally across all brain regions. For sociodemographic data, differences in continuous variables across groups were assessed with independent *t*-tests, while chi-square tests analyzed categorical interactions between groups.

We used linear models to explore SFC differences between older adults in the NC and DS groups, considering age, gender, education, and in-scanner head motion (root-mean-squared framewise displacement) as covariates. Effect sizes were determined using Cohen’s *d.*

Subgroup analyses compared SFC among five DS subgroups and NCs, adjusting for the same covariates mentioned above. We primarily focused on DS subgroups with or without diabetes, hyperlipidemia, hypertension, cerebral vascular disease, and sleep disorders. Variations within these subgroups were quantified using partial eta squared, and significant findings were further tested with post hoc 2-tailed t-tests, estimating effect sizes with Cohen’s *d* from the estimated marginal means.

Additionally, we fitted generalized additive models (GAMs) for each brain region to characterize how SFC changes with depressive symptoms, using GDS scores, and including the same covariates mentioned above. These GAMs estimated a smooth function describing the SFC-GDS relationship and its trajectory, with the derivative indicating changes at specific GDS scores (Supplementary Material).

Spin-based permutation testing was employed to assess the distributions of between-group differences and symptom-related effects across the cerebral cortex (Supplementary Material).

## Results

### Alterations of SFC in older adults with DS compared to NCs

**Figure 2.**
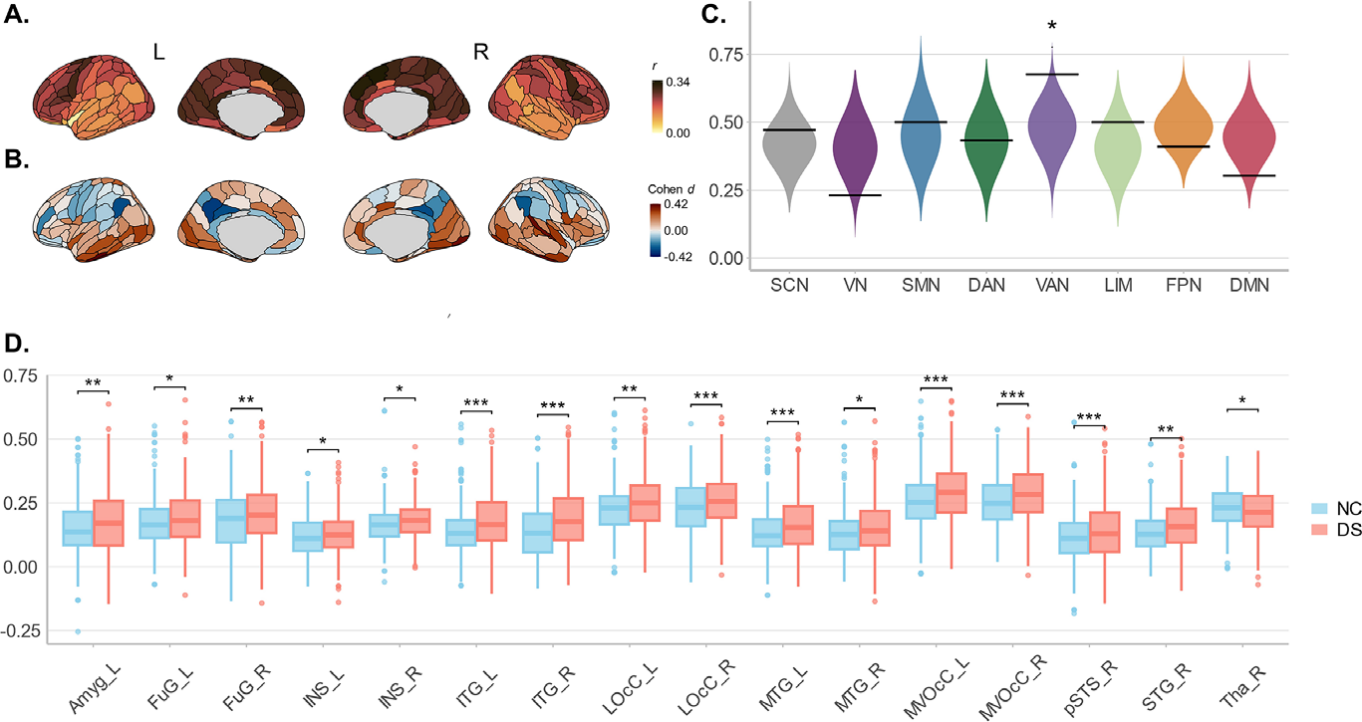
Differences in SFC between depressive symptom (DS) and normal control (NC) older adults. (a) Mean SFC of NCs. (b) SFC differences between DS and NC groups at each brain region. (c) Group differences between DS and NC older adults were enriched in the VAN, with 67.6% regions showing significant symptom-related changes via spin-based permutation test (*p* = .01). (D) Group differences between DS and NC older adults at the anatomical level. *Note*: Violins represent null distributions of test statistics; horizontal lines in violin plots, empirical test statistics; The upper and lower bounds of the boxes represent the first and third quartile, respectively; horizontal lines, median values; whiskers, 1.5× of upper and lower bounds of IQRs; and circles above and below boxes, outliers. SCN, ‘indicates subcortical network’; VN, ‘Visual network’; SMN, ‘somatosensory network’; DAN, ‘indicates dorsal attention network’; VAN, ‘ventral attention network’; LIM, ‘limbic network’; FPN, ‘frontoparietal network’; DMN, ‘default mode network’. DS, ‘depressive symptoms’; NC, ‘normal controls’. * *p* < .05; ^**^ *p* < .01; ^***^ *p* < .001.

We found wide variations in SFC across the brain (Figure 2a). The highest average SFC occurred in the right medial superior frontal gyrus (0.338 ± 0.006) and the lowest in the left ventral insula (0.019 ± 0.005). Compared with normal controls, older adults with depressive symptoms exhibited significantly increased SFC at 66 regions, and decreased SFC at 18 regions (Figure 2b). Increased regions were primarily involved in the ventral attention network (VAN), whereas decreased regions were primarily involved in the subcortical area and default mode network (DMN). Spin-based enrichment analysis found that regions with significant group differences tended to concentrate in VAN (67.6%, *p* = 0.01; Figure 2c), and all showed significantly increased SFC in the DS group.

The mean SFC of 48 anatomical structures (24 per hemisphere) was also compared between the NC and DS groups (Figure 2d). We found significantly increased mean SFC in 15 structural regions in the DS group, notably including the left amygdala, right superior temporal gyrus, and bilateral fusiform gyrus, while a significant decrease was observed in the right thalamus.

### Alterations of SFC in subgroups of older adults with depressive symptoms

**Figure 3.**
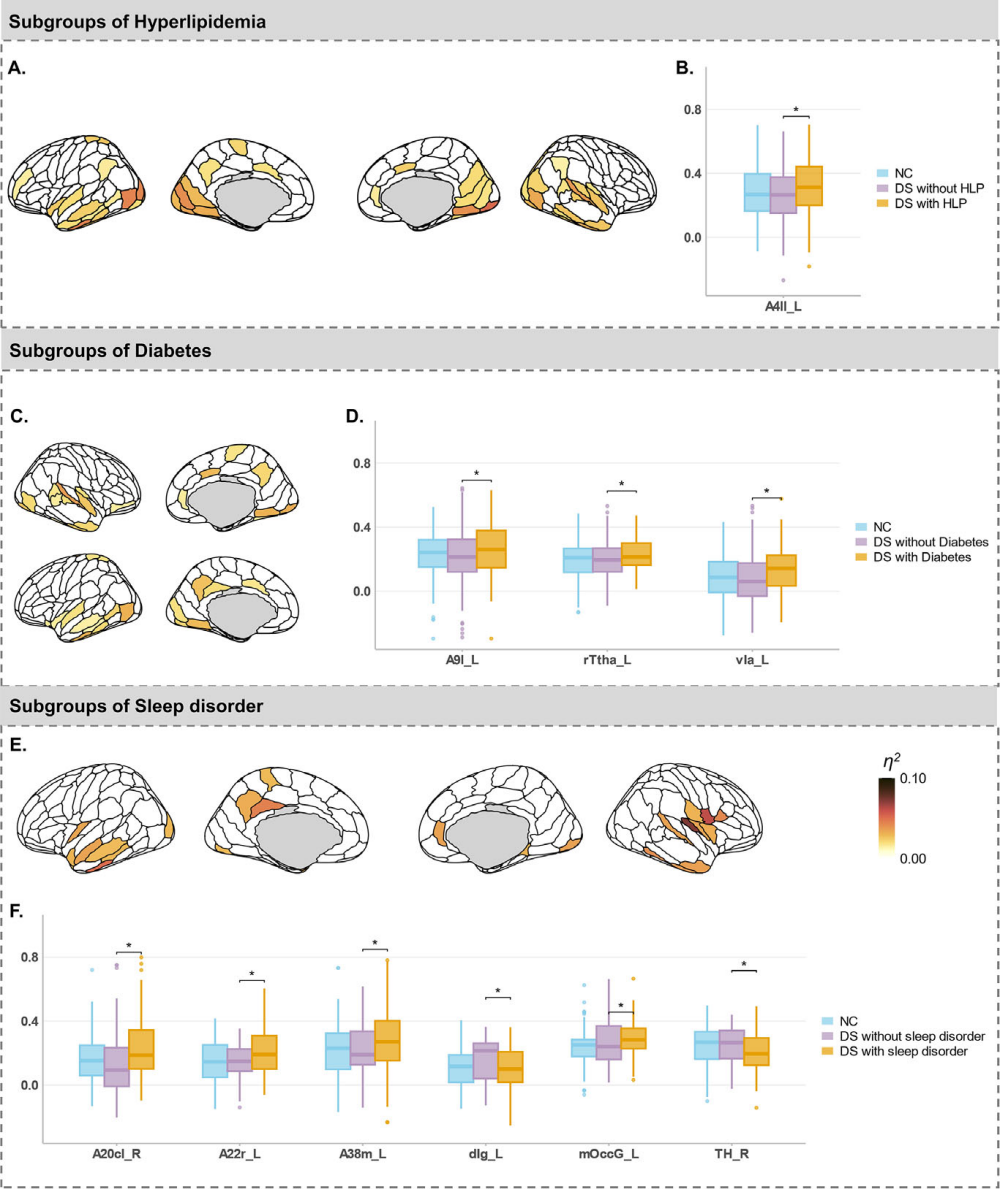
Differences in SFC among subgroups of older adults with depressive symptoms. (a) Regions with significant differences in SFC among NCs and DS older adults with and without diabetes. (b) Post-hoc comparisons of SFC between NCs and DS older adults with and without diabetes. (c) Regions with significant differences in SFC among NCs and DS older adults with and without hyperlipidemia. (d) Post-hoc comparisons of SFC between NCs and DS older adults with and without hyperlipidemia. (e) Regions with significant differences in SFC among NCs and DS older adults with and without sleep disorders. (f) Post-hoc comparisons of SFC between NCs and DS older adults with and without sleep disorders. *Note*: The upper and lower bounds of the boxes represent the first and third quartiles, respectively; horizontal lines, median values; whiskers, 1.5 × of the upper and lower bounds of IQRs; and circles above and below boxes, outliers. Partial *η^2^* of analysis of variance was mapped on the brain cerebral cortex, thresholding at false discovery rate–corrected *p* < .05. DS, ‘depressive symptoms’; NC, ‘normal controls’; HLP, ‘hyperlipidemia’. * *p* < .05.

To investigate the SFC changes associated with different physiological factors, we grouped participants by four chronic diseases (hypertension, hyperlipidemia, diabetes, and cerebral vascular disease) and a sleep disorder (Figure 3 and Supplementary Figure S1). As expected, we observed consistent SFC increases across multiple brain regions in all DS subgroups. Notably, DS-specific SFC alterations were detected primarily in the left hemisphere, involving areas such as the insula, frontal gyrus, and parahippocampus.

Specifically, compared with DS older adults without hyperlipidemia and normal controls, those with hyperlipidemia exhibited unique SFC increases in regions of the left paracentral lobe (partial *η* ^2^ = 0.022, 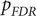 = 0.02). Compared with DS older adults without diabetes and normal controls, those with diabetes showed unique SFC increases in the left lateral superior frontal gyrus (partial *η* ^2^ = 0.014, 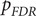 = 0.04), left ventral insula (partial *η* ^2^ = 0.017, 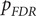 = 0.04) and left rostral thalamus (partial *η* ^2^ = 0.017, 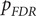 = 0.01). Subgroup-specific variations of SFC were most prominent in DS subgroups with sleep disorder, whereby sleep disorder was associated with increased SFC in the right parahippocampal gyrus (partial *η* ^2^ = 0.031, 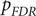 = 0.04) and left dorsal insula gyrus (partial *η* ^2^ = 0.035, 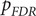 = 0.04) and decreased SFC in the left superior temporal gyrus (partial *η* ^2^ = 0.028, 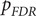 = 0.04; partial *η* ^2^ = 0.034, 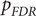 = 0.01), right inferior temporal gyrus (partial *η* ^2^ = 0.041, 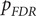 = 0.01) and left lateral middle occipital gyrus (partial *η* ^2^ = 0.028, 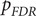 = 0.04). Collectively, these results suggest that SFC delineates subgroup differences precisely in older adults with DS and complex clinical profiles.

### Depressive symptoms-related effect on regional SFC

**Figure 4.**
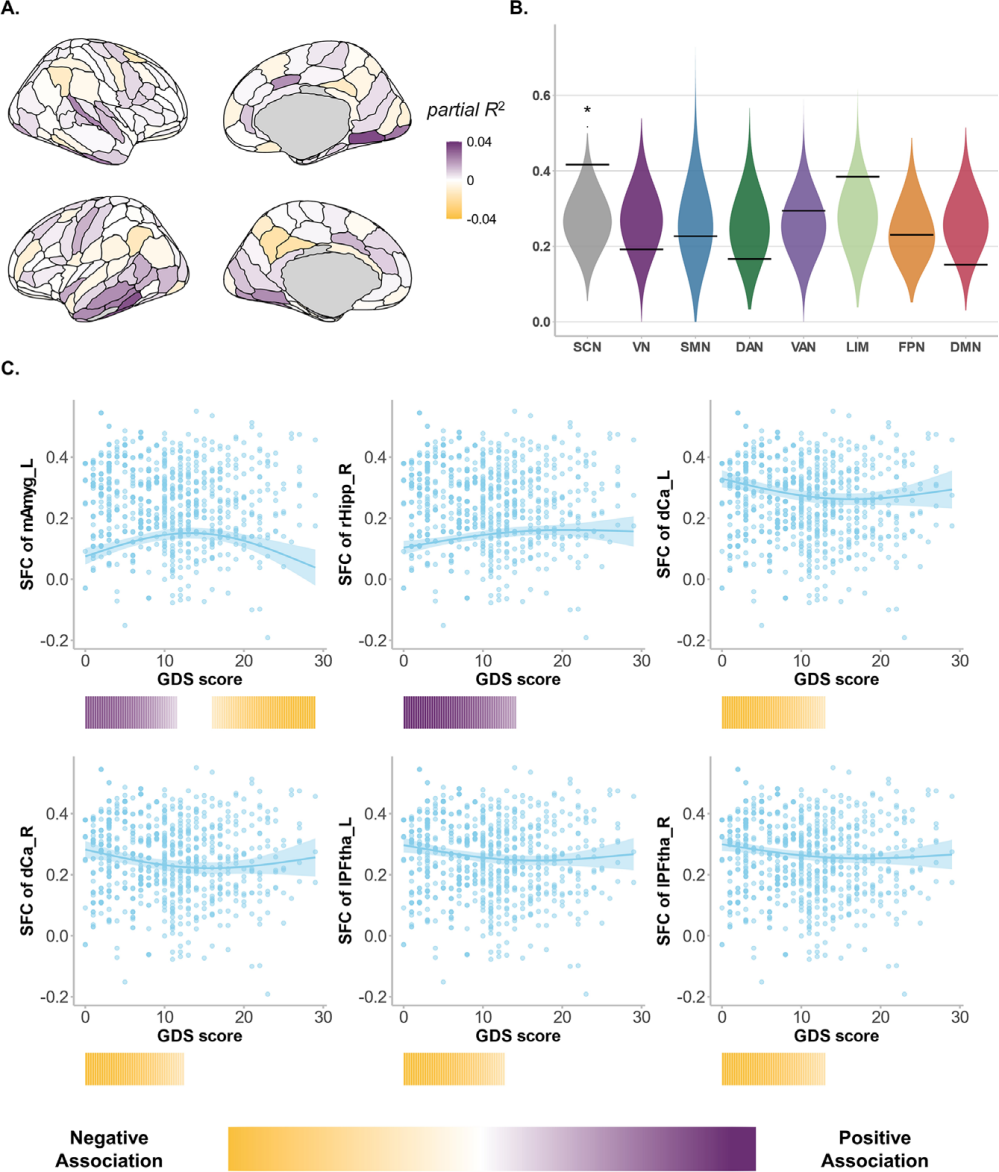
Associations between SFC and GDS scores. (a) regional SFC showed a significant effect (partial *R* ^2^) during progression of depressive symptoms as displayed across the cortical surface, thresholding at false discovery rate–corrected *p* < .05. (b) Depressive symptom-related effect was enriched in the SCN, with 41.7% regions showing significant symptom-related changes via spin-based permutation test (*p* = .02). (c) Symptom progression-related trajectories in SFC (zero-centred GAM smooth functions) are shown overlaid on data from all participants for all regions within the SCN that significantly changed as the symptom progressed. Regional trajectories represent the GAM-predicted SFC value at each GDS score point with a 95% credible interval band. The colour bars below each regional plot depict the GDS score window(s) wherein SFC significantly changed in that region, shaded by the rate of change, as determined by the first derivative of the age function. Windows of significant symptom-related change are alteration periods wherein the simultaneous 95% confidence interval for the first derivative did not include 0 (two-sided). The colour bar below represents the rate of change in SFC, with purple indicating a positive association and yellow indicating a negative one, where deeper colours signify stronger associations. *Note*: Violins represent null distributions of test statistics; horizontal lines in violin plots, empirical test statistics; The upper and lower bounds of the boxes represent the first and third quartile, respectively; horizontal lines, median values; whiskers, 1.5 × of upper and lower bounds of IQRs; and circles above and below boxes, outliers. SCN, ‘indicates subcortical network’; VN, ‘visual network’; SMN, ‘somatosensory network’; DAN, ‘indicates dorsal attention network’; VAN, ‘ventral attention network’; LIM, ‘limbic network’; FPN, ‘frontoparietal network’; DMN, ‘default mode network’; SFC, ‘structural functional coupling’; GDS, ‘geriatric depression scale’. * *p* < .05.

Regional SFC significantly changed with depressive symptom progression (i.e. GDS score) in 35 regions. The magnitude and direction of overall symptom-related effects differed across the cortex (Figure 4), signifying that there is variability in the symptom-related trajectories of SFC across the cerebral cortex. Then, by visualizing GDS score fits across these regions with significant symptom-related changes, we observed a continuum of symptom progression trajectories ranging from large and prolonged decreases to inverted *U*-shaped curves (Figure 4c). Spin-based enrichment analysis found that regions with significant symptom-related changes tended to concentrate in the subcortical area (Figure 4b, 41.7%, *p* = 0.02). Specifically, based on change rates of specific GDS score points (Figure 4c), we observed evident severity-dependent changes among all subcortical regions with significant symptom-related effects, mostly before GDS scores reached cutoff standards. Among these regions, the left amygdala stood out, showing a strong inverted *U*-shaped relationship with symptom severity: SFC increased at lower-to-moderate GDS scores but decreased at higher GDS scores.

### Sensitivity analysis

To assess the robustness of our findings and examine the potential confounding effects of hypertension and CVD, which were more prevalent in the DS group (Table 1), we conducted a sensitivity analysis. Specifically, we repeated the group differences analyses while including these comorbid conditions as covariates (Supplementary Figure S2). The results revealed that the different patterns of SFC remain consistent, with no significant changes compared to the main analyses.

## Discussion

We pioneered the use of multimodal neuroimaging to study structural–functional connectivity coupling (SFC) changes in older adults with depressive symptoms. Our study yielded three key findings. First, we observed increased SFC in older adults with depressive symptoms, particularly within the VAN. Second, subgroup analyses revealed specific SFC alterations associated with metabolic dysregulation and sleep disorders. Finally, we demonstrated that depressive symptom-related effects were primarily enriched in the SCN, with changes most pronounced at lower-to-moderate symptom severity levels, and SFC alterations in the left amygdala showed a consistent association with overall symptom burden. These findings filled a critical gap in our understanding of how white-matter architecture influences neural activity during the progression of depressive symptoms in older adults, providing neurobiological insight into the timely screening and management of depression among older adults.

### Increased SFC in older adults with depressive symptoms

We observed disrupted SFC patterns in older adults with depressive symptoms, primarily characterized by an increase across key networks, including the VN, VAN, LIM, and DMN. Notably, a consistent SFC increase was evident in the VAN, suggesting that the structural pathways more rigidly constrain functional communications in these regions, reducing their functional adaptability and flexibility (Baum et al., 2020; Medaglia et al., 2018; Paquola et al., 2019; Vázquez-Rodríguez et al., 2019). This is distinct from findings in younger populations with depression, where increased SFC was observed in the DMN (Chu et al., 2024; Jiang et al., 2019; Xu et al., 2024). These age-related differences may reflect distinct underlying mechanisms: in younger patients, DMN hyper-coupling may be linked to excessive self-referential processing and emotional dysregulation, whereas in older adults, VAN hyper-coupling could indicate compensatory shifts to support attentional function in the context of declining DMN integrity. Such divergence underscores the importance of considering both developmental stage and network-specific vulnerabilities when interpreting SFC changes in depression.

It is noteworthy that the prevalence of hypertension and CVD was significantly higher in the DS group than in the NC group. Given this, we conducted supplementary analyses to assess the potential confounding effects of these comorbidities. The additional results recapitulated the main effect: the DS group showed predominantly increased SFC within the VAN. This consistent pattern suggests that the maladaptive communication observed in the VAN in older adults with depressive symptoms is unlikely to be a consequence of microvascular damage. Rather, it might appear to reflect certain dysfunction of the intrinsic network (Alexopoulos et al., 2012; Csukly et al., 2024; Jiang et al., 2025; Kaiser, Andrews-Hanna, Wager, & Pizzagalli, 2015; Touron et al., 2022). Furthermore, subsequent subgroup analyses demonstrated consistently that neither hypertension nor CVD exerted specific effects on SFC in older adults with depressive symptoms.

Notably, the largest increases in SFC were identified in the bilateral ITG, both at the subregion and anatomical structure level. The ITG is critical for visual information processing and multimodal integration (Onitsuka et al., 2004). It receives input from the primary visual cortex and further projects to the amygdala-hippocampus circuit, essential for emotional and memory processing (Kravitz, Saleem, Baker, Ungerleider, & Mishkin, 2013). Thus, we can speculate that an abnormal increase in SFC in the ITG may potentially contribute to dysfunction of the amygdala-hippocampus circuit and could impair older adults’ abilities to flexibly process and integrate visual information in emotionally salient contexts.

### SFC alterations in subgroups of older adults with depressive symptoms

We identified subgroup-specific changes in SFC among subgroups of hyperlipidemia, diabetes, and sleep disorders, highlighting the heterogeneous neurobiological changes in older adults with depressive symptoms. Specifically, metabolic disease subgroups exhibited increased SFC in the left hemisphere, including the left paracentral lobe, lateral superior frontal gyrus, ventral insula, and rostral thalamus. This aligns with research suggesting a bidirectional relationship between metabolic dysregulation and depression, where metabolic issues can trigger neuroinflammatory processes and neurotransmitter system alterations, exacerbating depressive symptoms (Pan et al., 2012; Zhang, Chen, Yin, Wang, & Peng, 2021). Additionally, older adults with depressive symptoms and sleep disorders showed unique SFC patterns, with increases notably in the right parahippocampal gyrus and left dorsal insula, areas crucial to the frontal-limbic circuit responsible for emotion regulation. The frontal-limbic circuit plays a central role in the psychopathology of depression (Csukly et al., 2024; Qi et al., 2018), emphasizing the significant impact of sleep quality on frontal-limbic function(Plante, 2021). Conversely, decreased SFC in sensory processing areas such as the left superior temporal gyrus, right inferior temporal gyrus, and left lateral middle occipital gyrus suggests impairments in sensory integration, highlighting the complex interplay between sleep disturbances, sensory deficits, and depression (Nielson, Kay, & Dzierzewski, 2023; Tempesta, Socci, De Gennaro, & Ferrara, 2018).

These findings underscore the importance of considering the varied neuropathological profiles when studying depressive symptoms in older adults. Future research should integrate multimodal brain data with clinical factors like medication status and comorbidities to better understand this heterogeneity. Moreover, our results highlight the necessity of managing metabolic and sleep disorders within a comprehensive treatment approach for depression in older adults, as these conditions may exacerbate neurobiological vulnerabilities and influence treatment effectiveness.

### Disrupted SFC pattern along with depressive symptomatology among older adults

We observed that several regions showed symptom-related changes in SFC, and were enriched in the SCN, with the most pronounced effect occurring at lower severity levels. These severity-dependent changes may reflect neurobiological signatures of increasing symptom burden in older adults. When faced with structural damage related to pathological processes, especially, the brain may adopt adaptive reconfiguration strategies to maintain functional stability (Fotiadis et al., 2024; Gomez-Isla & Frosch, 2022). However, different regions within the SCN adopt distinct strategies to cope with these pre-abnormal symptoms. For instance, the left amygdala and right hippocampus showed increased SFC as the GDS score rises before reaching critical thresholds of abnormality. The increased coupling in these regions prior to pronounced depressive symptoms may try to strengthen reliance on existing structural pathways to preserve stability in crucial functions such as emotional perception and regulation (Lindquist, Wager, Kober, Bliss-Moreau, & Barrett, 2012). By contrast, decoupling in the bilateral caudal and thalamus emerged when facing pre-abnormal depressive symptomatic processes.

As symptoms progressed, only the SFC in the left amygdala continued to change, following an inverted *U*-shaped trajectory. This suggests that while there is an initial increased reliance on structural architecture to maintain functional organization, later stages of worsening symptoms are associated with increasing decoupling. The left amygdala plays a critical role in emotional regulation and response to stress (Kragel & LaBar, 2016; Sladky, Kargl, Haubensak, & Lamm, 2024), and its continuing altered coupling indicates a dynamic response to symptom severity of depression. As symptoms worsen, decoupling in left amygdala may be linked to neurotransmitter imbalances. Previous studies have indicated that decreased SFC in depressed patients can result from reductions in certain neurotransmitters that are most enriched in pathways of excitatory and inhibitory neurons (Berridge, 2017; Chu et al., 2024). Such imbalances can lead to decreased SFC, disrupting both structural and functional connectivity in neural circuits responsible for emotional regulation (Sears & Hewett, 2021), particularly those involving the left amygdala.

This study elucidates neurobiological mechanisms of SFC changes in older adults with depressive symptoms, showing increased SFC in the VAN network, which suggests impaired emotional regulation and attention. Subgroup analyses indicate that hyperlipidemia, diabetes, and sleep disorders distinctly affect depressive symptoms, underscoring their role in neurobiological vulnerabilities. Notably, SFC changes in the left amygdala could indicate symptom progression. These findings on the disrupted SFC patterns linked to depression in older adults could facilitate timely screening and effective interventions of depressive symptoms, thereby enhancing the clinical management of depression in older adults.

## Supporting information

Li et al. supplementary material Li et al. supplementary material

## Supplementary material

The supplementary material for this article can be found at http://doi.org/10.1017/S0033291725101657.
