# Supplementary material for: Alterations of structural–functional connectivity coupling in older adults with depressive symptoms
Source: Psychol Med. 2025 Oct 3;55:e293. doi: 10.1017/S0033291725101657 (PMC12527525; doi:10.1017/S0033291725101657)
Supplement: Li et al. supplementary material [file S0033291725101657sup001.docx]

Supplementary Materials

# Supplementary Methods

## Depressive symptom assessment

We assessed depressive symptoms using the self-reported Geriatric Depression Scale(Yesavage et al., 1982). This questionnaire ranges from 0 to 30, with higher scores indicating the presence of more depressive symptoms. Based on the GDS scores, participants were classified into two groups: those with depressive symptoms (Depressive Symptom group, DS, GDS ≥ 10) and those without (Normal Control group, NC, GDS < 10). This threshold was selected following the standard cutoff of the GDS (Yesavage et al., 1982).

## Image Acquisition

With the head snugly fixed by straps and foam pads, participants were asked to refrain from head movement. The functional images were acquired using an echo-planar imaging (EPI) sequence as follows: 33 axial slices, repetition time (TR) = 2000 ms, echo time (TE) = 30 ms, slice thickness = 3.5 mm, flip angle = 90°, field of view = 200 mm × 200 mm, acquisition matrix = 64 × 64, 240 volumes. The T1-weightedstructural data were also collected for spatial normalization using three-dimensional (3D) magnetization pre-pared rapid gradient echo (MP-RAGE) sequences as follows: 176 sagittal slices, repetition time = 1900 ms, echo time = 3.44 ms, slice thickness = 1 mm, flip angle = 9°, field of view = 200 mm × 200 mm, acquisition matrix = 256 × 256. DTI was acquired using a single shot, twice-refocused, diffusion-weighted echo planar imaging sequence coverage of the whole brain, 2-mm slice thickness with no interslice gap, 75 axial slices, TR = 8 000 ms, TE = 60 ms, FOA = 60°, acquisition matrix = 128 × 128, 30 diffusion directions with b = 1 000 s/mm2, and an additional image without diffusion weighting.

## Construction of the Functional Connectomes

fMRIPrep was additionally used to estimate the following 36 confounds from the preprocessed time series: six head motion parameters, three global signals (mean cerebrospinal fluid, white matter and whole-brain signals), temporal derivatives of the six head motion parameters and the three global signal estimates and quadratic terms for the motion parameters, tissue signals and their temporal derivatives. These confound matrices were used within xcp_d 0.0.4, which is an extension of the top-performing eXtensible Connectivity Pipeline (XCP) Engine specifically developed to mitigate motion-related artifacts and noise in resting-state fMRI data from developmental samples. With xcp_d, preprocessed functional time series on the fsLR cortical surface underwent nuisance regression using the 36 confounds listed above. Confounds were regressed using linear regression as implemented in Scikit-Learn (0.24.2).

## Generalized addictive model

To characterize changes in SFC related to depressive symptoms of each brain region, we fitted region-specific generalized additive models (GAMs) with a smooth term for GDS score; age, gender, education, and in-scanner head motion were included as linear covariates. Each GAM estimates a smooth function (the GDS model fit) that describes the relationship between regional SFC and GDS score, modeling the trajectory of depressive symptom progression of each region. The first derivative of this smooth function represents the rate of change in regional SFC at specific GDS score points.

GAMs were fitted with regional SFC as the dependent variable, GDS as a smooth term, and age, gender, education, and head motion as linear covariates. The models were applied separately for each parcellated brain region, using thin plate regression splines as the smooth term basis set and the restricted maximal likelihood approach for smoothing parameter selection. The smooth term for GDS in the GAM produced a spline, or a smooth function derived from a linear combination of weighted basis functions, representing each region’s trajectory of depressive symptom progression. To prevent overfitting, we set the maximum basis complexity (k) to 3, limiting the number of basis functions used to estimate the model fit. A value of *k* = 3 was chosen over higher values (e.g., *k* = 4–6) because it resulted in the lowest model Akaike information criterion for many cortical regions and was found to appropriately capture the depressive symptom progression trend with a conservative number of spines. Statistical tests of the *k*-index (Wood, 2017), which estimate the degree of unaccounted for non-random pattern in the residuals, confirmed that this basis dimension was sufficient.

For each regional generalized additive model (GAM), the significance of the association between regional SFC and GDS score was assessed by an analysis of variance (ANOVA), comparing the full GAM model to a nested, reduced model without GDS term. A significant result indicates that the residual deviance was significantly lower when a smooth term for GDS was included in the model, as determined by the chi-squared test statistic. ANOVA *P* values across all region-wise GAMs were corrected using the FDR correction and set statistical significance at *P*_FDR_ < 0.05. For each regional GAM with a significant GDS smooth term, we further identified the specific GDS score range(s) where SFC significantly changed by using the gratia package in R. The windows of significant change of the GDS scores were identified by calculating the first derivative of the GDS smooth function (Δ SFC/Δ GDS score) using finite differences and determining when the simultaneous 95% confidence interval of this derivative did not include 0 (Simpson, 2018). To quantify the overall magnitude and direction of the association between regional SFC and GDS (referred to as a region’s overall depressive symptom effect), we calculated the partial *R*^2^ between the full GAM model and the reduced model (effect magnitude) and signed the partial *R*^2^based on the sign of the average first derivative of the smooth function (effect direction).

## Enrichment analysis based on spin-based permutation testing

To evaluate whether the distribution of group differences and symptom-related associations tended to be concentrated within specific brain cortical networks, enrichment analysis was performed using spin−based permutation testing. The method is a conservative statistical approach that accounts for the varying sizes of cortical networks and the spatial autocorrelation of brain structures (www.github.com/frantisekvasa/rotate_parcellation)(Alexander-Bloch et al., 2018; Váša et al., 2018). Specifically, the proportion of cortical regions that survived the p-value threshold (*p* < .05, uncorrected) for group differences and symptom-related association analysis was calculated as the test statistics, respectively. The cortical statistical maps were projected onto a sphere and rotated 5000 times to create a null distribution of the test statistics(Baller et al., 2022). Networks were considered significantly enriched if the empirical test statistic fell within the top 5% of the null distribution, *i.e., p_spin_* < .05.

**Fig. S1. Differences in SFC among subgroups of older adults with depressive symptoms.**

**

**

**Fig. S1. Differences in SFC among subgroups of older adults with depressive symptoms. A.** Regions with significant differences in SFC among NCs, Hypertension- and Hypertension+. Partial *η^2^* is mapped on the brain, thresholding at FDR−corrected *p* < .05. **B.** Regions with significant differences in SFC among NCs, CVD- and CVD+. Partial *η^2^* is mapped on the brain, thresholding at FDR−corrected *p* < .05.

**Note.** SFC, structural-functional coupling; CVD, cerebral vascular disease.

# Fig. S2. Sensitivity analysis including hypertension and CVD as extra covariates.


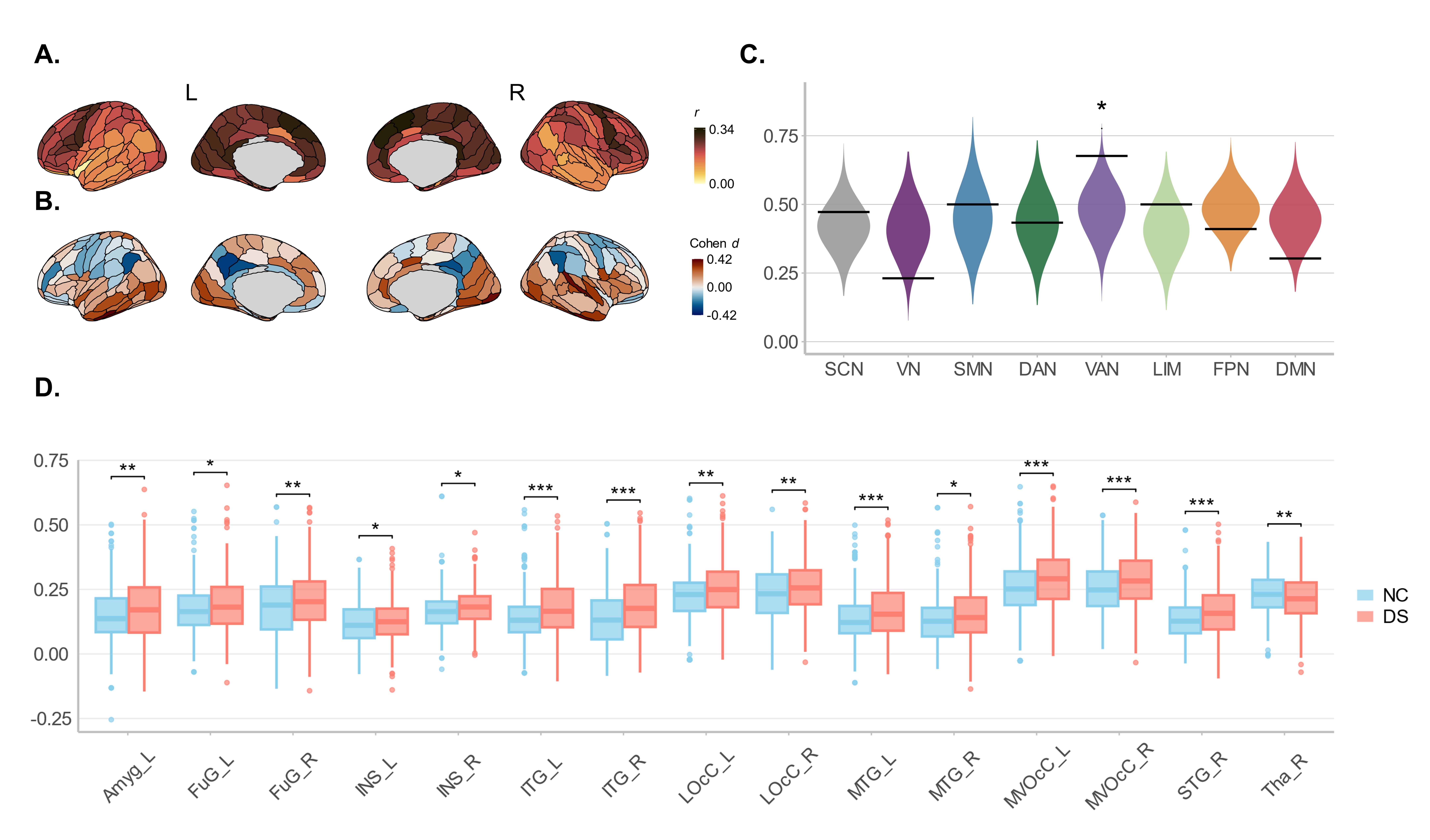


**Fig. S2. SFC differences between the DS and NC after controlling hypertension and CVD.** Confounders including Age, Gender, Education, Head motion, hypertension and CVD. **A.** Mean SFC of NCs. **B.** SFC differences between DS and NC groups at each brain region. **C.** Group differences between DS and NC older adults were enriched in the VAN, with 61.8% regions showed significant symptom-related changes via spin-based permutation test (*p* = .04). **D.** Group differences between DS and NC older adults at anatomical level.

**Note.** Violins represent null distributions of test statistics; horizontal lines in violin plots, empirical test statistics; The upper and lower bounds of the boxes represent the first and third quartile, respectively; horizontal lines, median values; whiskers, 1.5 × of upper and lower bounds of IQRs; and circles above and below boxes, outliers. SCN indicates subcortical network; VN, Visual network; SMN, somatosensory network; DAN indicates dorsal attention network; VAN, ventral attention network; LIM, limbic network; FPN, frontoparietal network; DMN, default mode network. DS, depressive symptoms; NC, normal controls.

^*^ *p* < .05; ^**^ *p* < .01; ^***^ *p* < .001.

**Table S1. Regions defined at different levels in the Brainnetome atlas**

| **Lobe** | **Gyrus** | **Left and Right Hemisphere** | **Label ID.L** | **Label ID.R** | **Anatomical and modified Cyto-architectonic descriptions** | **lh.MNI(X,Y,Z)** | **rh.MNI(X,Y,Z)** |
| --- | --- | --- | --- | --- | --- | --- | --- |
| **Frontal Lobe** | SFG, Superior Frontal Gyrus | SFG_L(R)_7_1 | 1 | 2 | *A8m, medial area 8* | -5 ,15, 54 | 7, 16, 54 |
|  |  | SFG_L(R)_7_2 | 3 | 4 | *A8dl, dorsolateral area 8* | -18, 24, 53 | 22, 26, 51 |
|  |  | SFG_L(R)_7_3 | 5 | 6 | *A9l, lateral area 9* | -11, 49, 40 | 13, 48, 40 |
|  |  | SFG_L(R)_7_4 | 7 | 8 | *A6dl, dorsolateral area 6* | -18, -1, 65 | 20, 4, 64 |
|  |  | SFG_L(R)_7_5 | 9 | 10 | *A6m, medial area 6* | -6, -5, 58 | 7, -4, 60 |
|  |  | SFG_L(R)_7_6 | 11 | 12 | *A9m,medial area 9* | -5, 36, 38 | 6, 38, 35 |
|  |  | SFG_L(R)_7_7 | 13 | 14 | *A10m, medial area 10* | -8, 56, 15 | 8, 58, 13 |
|  | MFG, Middle Frontal Gyrus | MFG_L(R)_7_1 | 15 | 16 | *A9/46d, dorsal area 9/46* | -27, 43, 31 | 30, 37, 36 |
|  |  | MFG_L(R)_7_2 | 17 | 18 | *IFJ, inferior frontal junction* | -42, 13, 36 | 42, 11, 39 |
|  |  | MFG_L(R)_7_3 | 19 | 20 | *A46, area 46* | -28, 56, 12 | 28, 55, 17 |
|  |  | MFG_L(R)_7_4 | 21 | 22 | *A9/46v, ventral area 9/46* | -41, 41, 16 | 42, 44, 14 |
|  |  | MFG_L(R)_7_5 | 23 | 24 | *A8vl, ventrolateral area 8* | -33, 23, 45 | 42, 27, 39 |
|  |  | MFG_L(R)_7_6 | 25 | 26 | *A6vl, ventrolateral area 6* | -32, 4, 55 | 34, 8, 54 |
|  |  | MFG_L(R)_7_7 | 27 | 28 | *A10l, lateral area10* | -26, 60, -6 | 25, 61, -4 |
|  | IFG, Inferior Frontal Gyrus | IFG_L(R)_6_1 | 29 | 30 | *A44d,dorsal area 44* | -46, 13, 24 | 45, 16, 25 |
|  |  | IFG_L(R)_6_2 | 31 | 32 | *IFS, inferior frontal sulcus* | -47, 32, 14 | 48, 35, 13 |
|  |  | IFG_L(R)_6_3 | 33 | 34 | *A45c, caudal area 45* | -53, 23, 11 | 54, 24, 12 |
|  |  | IFG_L(R)_6_4 | 35 | 36 | *A45r, rostral area 45* | -49, 36, -3 | 51, 36, -1 |
|  |  | IFG_L(R)_6_5 | 37 | 38 | *A44op, opercular area 44* | -39, 23, 4 | 42, 22, 3 |
|  |  | IFG_L(R)_6_6 | 39 | 40 | *A44v, ventral area 44* | -52, 13, 6 | 54, 14, 11 |
|  | OrG, Orbital Gyrus | OrG_L(R)_6_1 | 41 | 42 | *A14m, medial area 14* | -7, 54, -7 | 6, 47, -7 |
|  |  | OrG_L(R)_6_2 | 43 | 44 | *A12/47o, orbital area 12/47* | -36, 33, -16 | 40, 39, -14 |
|  |  | OrG_L(R)_6_3 | 45 | 46 | *A11l, lateral area 11* | -23, 38, -18 | 23, 36, -18 |
|  |  | OrG_L(R)_6_4 | 47 | 48 | *A11m, medial area 11* | -6, 52, -19 | 6, 57, -16 |
|  |  | OrG_L(R)_6_5 | 49 | 50 | *A13, area 13* | -10, 18, -19 | 9, 20, -19 |
|  |  | OrG_L(R)_6_6 | 51 | 52 | *A12/47l, lateral area 12/47* | -41, 32, -9 | 42, 31, -9 |
|  | PrG, Precentral Gyrus | PrG_L(R)_6_1 | 53 | 54 | *A4hf, area 4(head and face region)* | -49, -8, 39 | 55, -2, 33 |
|  |  | PrG_L(R)_6_2 | 55 | 56 | *A6cdl, caudal dorsolateral area 6* | -32, -9, 58 | 33, -7, 57 |
|  |  | PrG_L(R)_6_3 | 57 | 58 | *A4ul, area 4(upper limb region)* | -26, -25, 63 | 34, -19, 59 |
|  |  | PrG_L(R)_6_4 | 59 | 60 | *A4t, area 4(trunk region)* | -13, -20, 73 | 15, -22, 71 |
|  |  | PrG_L(R)_6_5 | 61 | 62 | *A4tl, area 4(tongue and larynx region)* | -52, 0, 8 | 54, 4, 9 |
|  |  | PrG_L(R)_6_6 | 63 | 64 | *A6cvl, caudal ventrolateral area 6* | -49, 5, 30 | 51, 7, 30 |
|  | PCL, Paracentral Lobule | PCL_L(R)_2_1 | 65 | 66 | *A1/2/3ll, area1/2/3 (lower limb region)* | -8, -38, 58 | 10, -34, 54 |
|  |  | PCL_L(R)_2_2 | 67 | 68 | *A4ll, area 4, (lower limb region)* | -4, -23, 61 | 5, -21, 61 |
| **Temporal Lobe** | STG, Superior Temporal Gyrus | STG_L(R)_6_1 | 69 | 70 | *A38m, medial area 38* | -32, 14, -34 | 31, 15, -34 |
|  |  | STG_L(R)_6_2 | 71 | 72 | *A41/42, area 41/42* | -54, -32, 12 | 54, -24, 11 |
|  |  | STG_L(R)_6_3 | 73 | 74 | *TE1.0 and TE1.2* | -50, -11, 1 | 51, -4, -1 |
|  |  | STG_L(R)_6_4 | 75 | 76 | *A22c, caudal area 22* | -62, -33, 7 | 66, -20, 6 |
|  |  | STG_L(R)_6_5 | 77 | 78 | *A38l, lateral area 38* | -45, 11, -20 | 47, 12, -20 |
|  |  | STG_L(R)_6_6 | 79 | 80 | *A22r, rostral area 22* | -55, -3, -10 | 56, -12, -5 |
|  | MTG, Middle Temporal Gyrus | MTG_L(R)_4_1 | 81 | 82 | *A21c, caudal area 21* | -65, -30, -12 | 65, -29, -13 |
|  |  | MTG_L(R)_4_2 | 83 | 84 | *A21r, rostral area 21* | -53, 2, -30 | 51, 6, -32 |
|  |  | MTG_L(R)_4_3 | 85 | 86 | *A37dl, dorsolateral area37* | -59, -58, 4 | 60, -53, 3 |
|  |  | MTG_L(R)_4_4 | 87 | 88 | *aSTS, anterior superior temporal sulcus* | -58, -20, -9 | 58, -16, -10 |
|  | ITG, Inferior Temporal Gyrus | ITG_L(R)_7_1 | 89 | 90 | *A20iv, intermediate ventral area 20* | -45, -26, -27 | 46, -14, -33 |
|  |  | ITG_L(R)_7_2 | 91 | 92 | *A37elv, extreme lateroventral area37* | -51, -57, -15 | 53, -52, -18 |
|  |  | ITG_L(R)_7_3 | 93 | 94 | *A20r, rostral area 20* | -43, -2, -41 | 40, 0, -43 |
|  |  | ITG_L(R)_7_4 | 95 | 96 | *A20il, intermediate lateral area 20* | -56, -16, -28 | 55, -11, -32 |
|  |  | ITG_L(R)_7_5 | 97 | 98 | *A37vl, ventrolateral area 37* | -55, -60, -6 | 54, -57, -8 |
|  |  | ITG_L(R)_7_6 | 99 | 100 | *A20cl, caudolateral of area 20* | -59, -42, -16 | 61, -40, -17 |
|  |  | ITG_L(R)_7_7 | 101 | 102 | *A20cv, caudoventral of area 20* | -55, -31, -27 | 54, -31, -26 |
|  | FuG, Fusiform Gyrus | FuG_L(R)_3_1 | 103 | 104 | *A20rv, rostroventral area 20* | -33, -16, -32 | 33, -15, -34 |
|  |  | FuG_L(R)_3_2 | 105 | 106 | *A37mv, medioventral area37* | -31, -64, -14 | 31, -62, -14 |
|  |  | FuG_L(R)_3_3 | 107 | 108 | *A37lv, lateroventral area37* | -42, -51, -17 | 43, -49, -19 |
|  | PhG, Parahippocampal Gyrus | PhG_L(R)_6_1 | 109 | 110 | *A35/36r, rostral area 35/36* | -27, -7, -34 | 28, -8, -33 |
|  |  | PhG_L(R)_6_2 | 111 | 112 | *A35/36c, caudal area 35/36* | -25, -25, -26 | 26, -23, -27 |
|  |  | PhG_L(R)_6_3 | 113 | 114 | *TL, area TL (lateral PPHC, posterior parahippocampal gyrus)* | -28, -32, -18 | 30, -30, -18 |
|  |  | PhG_L(R)_6_4 | 115 | 116 | *A28/34, area 28/34 (EC, entorhinal cortex)* | -19, -12, -30 | 19, -10, -30 |
|  |  | PhG_L(R)_6_5 | 117 | 118 | *TI, area TI(temporal agranular insular cortex)* | -23, 2, -32 | 22, 1, -36 |
|  |  | PhG_L(R)_6_6 | 119 | 120 | *TH, area TH (medial PPHC)* | -17, -39, -10 | 19, -36, -11 |
|  | pSTS, posterior Superior Temporal Sulcus | pSTS_L(R)_2_1 | 121 | 122 | *rpSTS, rostroposterior superior temporal sulcus* | -54, -40, 4 | 53, -37, 3 |
|  |  | pSTS_L(R)_2_2 | 123 | 124 | *cpSTS, caudoposterior superior temporal sulcus* | -52, -50, 11 | 57, -40, 12 |
| **Parietal Lobe** | SPL, Superior Parietal Lobule | SPL_L(R)_5_1 | 125 | 126 | *A7r, rostral area 7* | -16, -60, 63 | 19, -57, 65 |
|  |  | SPL_L(R)_5_2 | 127 | 128 | *A7c, caudal area 7* | -15, -71, 52 | 19, -69, 54 |
|  |  | SPL_L(R)_5_3 | 129 | 130 | *A5l, lateral area 5* | -33, -47, 50 | 35, -42, 54 |
|  |  | SPL_L(R)_5_4 | 131 | 132 | *A7pc, postcentral area 7* | -22, -47, 65 | 23, -43, 67 |
|  |  | SPL_L(R)_5_5 | 133 | 134 | *A7ip, intraparietal area 7(hIP3)* | -27, -59, 54 | 31, -54, 53 |
|  | IPL, Inferior Parietal Lobule | IPL_L(R)_6_1 | 135 | 136 | *A39c, caudal area 39(PGp)* | -34, -80, 29 | 45, -71, 20 |
|  |  | IPL_L(R)_6_2 | 137 | 138 | *A39rd, rostrodorsal area 39(Hip3)* | -38, -61, 46 | 39, -65, 44 |
|  |  | IPL_L(R)_6_3 | 139 | 140 | *A40rd, rostrodorsal area 40(PFt)* | -51, -33, 42 | 47, -35, 45 |
|  |  | IPL_L(R)_6_4 | 141 | 142 | *A40c, caudal area 40(PFm)* | -56, -49, 38 | 57, -44, 38 |
|  |  | IPL_L(R)_6_5 | 143 | 144 | *A39rv, rostroventral area 39(PGa)* | -47, -65, 26 | 53, -54, 25 |
|  |  | IPL_L(R)_6_6 | 145 | 146 | *A40rv, rostroventral area 40(PFop)* | -53, -31, 23 | 55, -26, 26 |
|  | Pcun, Precuneus | PCun_L(R)_4_1 | 147 | 148 | *A7m, medial area 7(PEp)* | -5, -63, 51 | 6, -65, 51 |
|  |  | PCun_L(R)_4_2 | 149 | 150 | *A5m, medial area 5(PEm)* | -8, -47, 57 | 7, -47, 58 |
|  |  | PCun_L(R)_4_3 | 151 | 152 | *dmPOS, dorsomedial parietooccipital sulcus(PEr)* | -12, -67, 25 | 16, -64, 25 |
|  |  | PCun_L(R)_4_4 | 153 | 154 | *A31, area 31 (Lc1)* | -6, -55, 34 | 6, -54, 35 |
|  | PoG, Postcentral Gyrus | PoG_L(R)_4_1 | 155 | 156 | *A1/2/3ulhf, area 1/2/3(upper limb, head and face region)* | -50, -16, 43 | 50, -14, 44 |
|  |  | PoG_L(R)_4_2 | 157 | 158 | *A1/2/3tonIa, area 1/2/3(tongue and larynx region)* | -56, -14, 16 | 56, -10, 15 |
|  |  | PoG_L(R)_4_3 | 159 | 160 | *A2, area 2* | -46, -30, 50 | 48, -24, 48 |
|  |  | PoG_L(R)_4_4 | 161 | 162 | *A1/2/3tru, area1/2/3(trunk region)* | -21, -35, 68 | 20, -33, 69 |
| **Insular Lobe** | INS, Insular Gyrus | INS_L(R)_6_1 | 163 | 164 | *G, hypergranular insula* | -36, -20, 10 | 37, -18, 8 |
|  |  | INS_L(R)_6_2 | 165 | 166 | *vIa, ventral agranular insula* | -32, 14, -13 | 33, 14, -13 |
|  |  | INS_L(R)_6_3 | 167 | 168 | *dIa, dorsal agranular insula* | -34, 18, 1 | 36, 18, 1 |
|  |  | INS_L(R)_6_4 | 169 | 170 | *vId/vIg, ventral dysgranular and granular insula* | -38, -4, -9 | 39, -2, -9 |
|  |  | INS_L(R)_6_5 | 171 | 172 | *dIg, dorsal granular insula* | -38, -8, 8 | 39, -7, 8 |
|  |  | INS_L(R)_6_6 | 173 | 174 | *dId, dorsal dysgranular insula* | -38, 5, 5 | 38, 5, 5 |
| **Limbic Lobe** | CG, Cingulate Gyrus | CG_L(R)_7_1 | 175 | 176 | *A23d, dorsal area 23* | -4, -39, 31 | 4, -37, 32 |
|  |  | CG_L(R)_7_2 | 177 | 178 | *A24rv, rostroventral area 24* | -3, 8, 25 | 5, 22, 12 |
|  |  | CG_L(R)_7_3 | 179 | 180 | *A32p, pregenual area 32* | -6, 34, 21 | 5, 28, 27 |
|  |  | CG_L(R)_7_4 | 181 | 182 | *A23v, ventral area 23* | -8, -47, 10 | 9, -44, 11 |
|  |  | CG_L(R)_7_5 | 183 | 184 | *A24cd, caudodorsal area 24* | -5, 7, 37 | 4, 6, 38 |
|  |  | CG_L(R)_7_6 | 185 | 186 | *A23c, caudal area 23* | -7, -23, 41 | 6, -20, 40 |
|  |  | CG_L(R)_7_7 | 187 | 188 | *A32sg, subgenual area 32* | -4, 39, -2 | 5, 41, 6 |
| **Occipital Lobe** | MVOcC*,* MedioVentral Occipital Cortex | MVOcC _L(R)_5_1 | 189 | 190 | *cLinG, caudal lingual gyrus* | -11, -82, -11 | 10, -85, -9 |
|  |  | MVOcC _L(R)_5_2 | 191 | 192 | *rCunG, rostral cuneus gyrus* | -5, -81, 10 | 7, -76, 11 |
|  |  | MVOcC _L(R)_5_3 | 193 | 194 | *cCunG, caudal cuneus gyrus* | -6, -94, 1 | 8, -90, 12 |
|  |  | MVOcC _L(R)_5_4 | 195 | 196 | *rLinG, rostral lingual gyrus* | -17, -60, -6 | 18, -60, -7 |
|  |  | MVOcC _L(R)_5_5 | 197 | 198 | *vmPOS,ventromedial parietooccipital sulcus* | -13, -68, 12 | 15, -63, 12 |
|  | LOcC, lateral Occipital Cortex | LOcC_L(R)_4_1 | 199 | 200 | *mOccG, middle occipital gyrus* | -31, -89, 11 | 34, -86, 11 |
|  |  | LOcC _L(R)_4_2 | 201 | 202 | *V5/MT+, area V5/MT+* | -46, -74, 3 | 48, -70, -1 |
|  |  | LOcC _L(R)_4_3 | 203 | 204 | *OPC, occipital polar cortex* | -18, -99, 2 | 22, -97, 4 |
|  |  | LOcC_L(R)_4_4 | 205 | 206 | *iOccG, inferior occipital gyrus* | -30, -88, -12 | 32, -85, -12 |
|  |  | LOcC _L(R)_2_1 | 207 | 208 | *msOccG, medial superior occipital gyrus* | -11, -88, 31 | 16, -85, 34 |
|  |  | LOcC _L(R)_2_2 | 209 | 210 | *lsOccG, lateral superior occipital gyrus* | -22, -77, 36 | 29, -75, 36 |
| **Subcortical Nuclei** | Amyg, Amygdala | Amyg_L(R)_2_1 | 211 | 212 | *mAmyg, medial amygdala* | -19, -2, -20 | 19, -2, -19 |
|  |  | Amyg_L(R)_2_2 | 213 | 214 | *lAmyg, lateral amygdala* | -27, -4, -20 | 28, -3, -20 |
|  | Hipp, Hippocampus | Hipp_L(R)_2_1 | 215 | 216 | *rHipp, rostral hippocampus* | -22, -14, -19 | 22, -12, -20 |
|  |  | Hipp_L(R)_2_2 | 217 | 218 | *cHipp, caudal hippocampus* | -28, -30, -10 | 29, -27, -10 |
|  | BG, Basal Ganglia | BG_L(R)_6_1 | 219 | 220 | *vCa, ventral caudate* | -12, 14, 0 | 15, 14, -2 |
|  |  | BG_L(R)_6_2 | 221 | 222 | *GP, globus pallidus* | -22, -2, 4 | 22, -2, 3 |
|  |  | BG_L(R)_6_3 | 223 | 224 | *NAC, nucleus accumbens* | -17, 3, -9 | 15, 8, -9 |
|  |  | BG_L(R)_6_4 | 225 | 226 | *vmPu, ventromedial putamen* | -23, 7, -4 | 22, 8, -1 |
|  |  | BG_L(R)_6_5 | 227 | 228 | *dCa, dorsal caudate* | -14, 2, 16 | 14, 5, 14 |
|  |  | BG_L(R)_6_6 | 229 | 230 | *dlPu, dorsolateral putamen* | -28, -5, 2 | 29, -3, 1 |
|  | Tha, Thalamus | Tha_L(R)_8_1 | 231 | 232 | *mPFtha, medial pre-frontal thalamus* | -7, -12, 5 | 7, -11, 6 |
|  |  | Tha_L(R)_8_2 | 233 | 234 | *mPMtha, pre-motor thalamus* | -18, -13, 3 | 12, -14, 1 |
|  |  | Tha_L(R)_8_3 | 235 | 236 | *Stha, sensory thalamus* | -18, -23, 4 | 18, -22, 3 |
|  |  | Tha_L(R)_8_4 | 237 | 238 | *rTtha, rostral temporal thalamus* | -7, -14, 7 | 3, -13, 5 |
|  |  | Tha_L(R)_8_5 | 239 | 240 | *PPtha, posterior parietal thalamus* | -16, -24, 6 | 15, -25, 6 |
|  |  | Tha_L(R)_8_6 | 241 | 242 | *Otha, occipital thalamus* | -15, -28, 4 | 13, -27, 8 |
|  |  | Tha_L(R)_8_7 | 243 | 244 | *cTtha, caudal temporal thalamus* | -12, -22, 13 | 10, -14, 14 |
|  |  | Tha_L(R)_8_8 | 245 | 246 | *lPFtha, lateral pre-frontal thalamus* | -11, -14, 2 | 13, -16, 7 |

**Table S2. Demographic information of the subset participants with the PSQI**

| Characteristic | Normal Control (n = 186) | Depressive Symptom (n = 272) | *t/χ^2^* | *p* |
| --- | --- | --- | --- | --- |
| **Demographic Information** |  |  |  |  |
| Age (years) | 66.22±5.95 | 66.44±6.41 | -0.372 | .710 |
| Gender (M/F) | 51/135 | 81/191 | 0.196 | .658 |
| Education (years) | 11.34±3.06 | 10.94±2.75 | 1.432 | .153 |
| Marriage  (married/ unmarried) | 144/42 | 220/52 | 3.184 | .672 |
| **Clinical Characteristics** |  |  |  |  |
| Hypertension | 75(40.32%) | 149(54.78%) | 11.444 | .003 |
| Hyperlipemia | 66 (35.48%) | 143 (52.57%) | 13.027 | .001 |
| Diabetes | 37 (19.89%) | 77 (28.31%) | 4.699 | .095 |
| Cerebral vascular disease | 25 (13.44%) | 90 (33.09%) | 23.552 | < .001 |
| Sleep Disorders | 77(41.40%) | 179(65.81%) | 25.720 | < .001 |

Note. This table present the demographic information of the subset of participants with complete Pittsburgh Sleep Quality Index (PSQI) data. Due to limitations in data collection time, participant consent, and data quality issues, only 458 out of 830 participants had PSQI data available for further subgroup analysis.

**Table S3. Demographic information of subgroups**

| **Demographic Information** | **Normal Control** | **Hypertension-** | **Hypertension+** | ***F/χ^2^*** | ***p*** |
| --- | --- | --- | --- | --- | --- |
|  | **(n = 238)** | **(n = 201)** | **(n = 210)** |  |  |
| Age (years) | 65.05±5.98 | 64.85±6.43 | 67.07±6.71 | 7.849 | < .001 |
| Gender (M/F) | 80/158 | 69/132 | 76/134 | 0.133 | .715 |
| Education (years) | 11.33±2.84 | 11.02±2.8 | 10.97±2.98 | 1.017 | .362 |
| Marriage (married/ unmarried) | 197/41 | 162/39 | 172/38 | 1.895 | .169 |
|  | **Normal Control** | **HLP-** | **HLP+** | ***F/χ^2^*** | ***p*** |
|  | **(n = 243)** | **(n = 217)** | **(n = 185)** |  |  |
| Age (years) | 66.15±6.23 | 65.2±6.8 | 66.78±6.45 | 3.033 | .049 |
| Gender (M/F) | 89/154 | 87/130 | 55/130 | 4.777 | .092 |
| Education (years) | 11.1±2.92 | 10.97±2.81 | 10.97±2.96 | 0.167 | .846 |
| Marriage (married/ unmarried) | 198/45 | 179/38 | 148/37 | 8.386 | .591 |
|  | **Normal Control** | **Diabetes-** | **Diabetes+** | ***F/χ^2^*** | ***p*** |
|  | **(n = 243)** | **(n = 289)** | **(n = 117)** |  |  |
| Age (years) | 65.71±6.18 | 65.73±6.73 | 66.81±6.35 | 1.403 | .247 |
| Gender (M/F) | 97/146 | 102/115 | 43/74 | 0.229 | .892 |
| Education (years) | 11.51±2.8 | 10.75±2.79 | 11.61±3.05 | 6.482 | .002 |
| Marriage (married/ unmarried) | 232/11 | 232/57 | 99/18 | 11.731 | .303 |
|  | **Normal Control** | **CVD-** | **CVD+** | ***F/χ^2^*** | ***p*** |
|  | **(n = 329)** | **(n = 276)** | **(n = 122)** |  |  |
| Age (years) | 65.47±6.07 | 65.43±6.69 | 67.16±6.45 | 3.663 | .026 |
| Gender (M/F) | 126/203 | 96/180 | 49/95 | 1.316 | .518 |
| Education (years) | 11.33±2.71 | 10.91±2.96 | 11.14±2.73 | 1.661 | .191 |
| Marriage (married/ unmarried) | 278/51 | 231/45 | 97/25 | 15.104 | .128 |
|  | **Normal Control** | **Sleep disorder-** | **Sleep disorder+** | ***F/χ^2^*** | ***p*** |
|  | **(n = 109)** | **(n = 93)** | **(n = 179)** |  |  |
| Age (years) | 66.11±6.01 | 66.38±5.9 | 65.61±5.72 | 0.377 | .686 |
| Gender (M/F) | 43/66 | 29/64 | 52/127 | 3.441 | .179 |
| Education (years) | 11.56±2.96 | 11.01±3.2 | 10.52±2.52 | 3.314 | .038 |
| Marriage (married/ unmarried) | 88/21 | 75/18 | 145/34 | 7.497 | .678 |

Note. This table presents demographic information categorized into subgroups based on five key physiological health factors: hypertension, hyperlipidemia, diabetes, cerebral vascular disease, and sleep disorders. Each health factor has corresponding subgroups including:

1. Normal controls without hypertension (NC) and older adults in the depressive group, classified based on hypertension status (Hypertension+/Hypertension-).

2. Normal controls without hyperlipidemia (NC) alongside older adults in the depressive group, classified based on hyperlipidemia status (HLP+/HLP-).

3. Normal controls without diabetes (NC) and older adults in the depressive group, distinguished by diabetes status (Diabetes+/Diabetes-).

4. Normal controls without cerebral vascular disease (NC) and older adults in the depressive group, classified based on cerebral vascular disease status (CVD+/CVD-).

5. Normal controls without sleep disorders (NC) and older adults in the depressive group, classified based on sleep disorder status measured by the PSQI, with a total score greater than 5 indicating a sleep disorder (Sleep disorder+/Sleep disorder-).

ANOVA was performed to analysis group differences of age and education level. 𝜒^2^ test was performed to analyze interactions between groups and categorical variables, including gender and marriage status.

**Table S4. The network definition in Brainnetome atlas.**

| ID | Description | Subregion | Network | ID | Description | Subregion | Network | |
| --- | --- | --- | --- | --- | --- | --- | --- | --- |
| 1 | A8m | SFG_L_7_1 | FPN | 2 | A8m | SFG_L_7_1 | FPN |  |
| 3 | A8dl | SFG_L_7_2 | DMN | 4 | A8dl | SFG_L_7_2 | DMN |  |
| 5 | A9l | SFG_L_7_3 | DMN | 6 | A9l | SFG_L_7_3 | DMN |  |
| 7 | A6dl | SFG_L_7_4 | DAN | 8 | A6dl | SFG_L_7_4 | DAN |  |
| 9 | A6m | SFG_L_7_5 | SMN | 10 | A6m | SFG_L_7_5 | SMN |  |
| 11 | A9m | SFG_L_7_6 | DMN | 12 | A9m | SFG_L_7_6 | DMN |  |
| 13 | A10m | SFG_L_7_7 | DMN | 14 | A10m | SFG_L_7_7 | DMN |  |
| 15 | A9/46d | MFG_L_7_1 | VAN | 16 | A9/46d | MFG_L_7_1 | VAN |  |
| 17 | IFJ | MFG_L_7_2 | FPN | 18 | IFJ | MFG_L_7_2 | FPN |  |
| 19 | A46 | MFG_L_7_3 | FPN | 20 | A46 | MFG_L_7_3 | FPN |  |
| 21 | A9/46v | MFG_L_7_4 | FPN | 22 | A9/46v | MFG_L_7_4 | FPN |  |
| 23 | A8vl | MFG_L_7_5 | DMN | 24 | A8vl | MFG_L_7_5 | DMN |  |
| 25 | A6vl | MFG_L_7_6 | DAN | 26 | A6vl | MFG_L_7_6 | DAN |  |
| 27 | A10l | MFG_L_7_7 | LIM | 28 | A10l | MFG_L_7_7 | LIM |  |
| 29 | A44d | IFG_L_6_1 | FPN | 30 | A44d | IFG_L_6_1 | FPN |  |
| 31 | IFS | IFG_L_6_2 | FPN | 32 | IFS | IFG_L_6_2 | FPN |  |
| 33 | A45c | IFG_L_6_3 | DMN | 34 | A45c | IFG_L_6_3 | DMN |  |
| 35 | A45r | IFG_L_6_4 | DMN | 36 | A45r | IFG_L_6_4 | DMN |  |
| 37 | A44op | IFG_L_6_5 | VAN | 38 | A44op | IFG_L_6_5 | VAN |  |
| 39 | A44v | IFG_L_6_6 | VAN | 40 | A44v | IFG_L_6_6 | VAN |  |
| 41 | A14m | OrG_L_6_1 | DMN | 42 | A14m | OrG_L_6_1 | DMN |  |
| 43 | A12/47o | OrG_L_6_2 | DMN | 44 | A12/47o | OrG_L_6_2 | DMN |  |
| 45 | A11l | OrG_L_6_3 | LIM | 46 | A11l | OrG_L_6_3 | LIM |  |
| 47 | A11m | OrG_L_6_4 | LIM | 48 | A11m | OrG_L_6_4 | LIM |  |
| 49 | A13 | OrG_L_6_5 | LIM | 50 | A13 | OrG_L_6_5 | LIM |  |
| 51 | A12/47l | OrG_L_6_6 | DMN | 52 | A12/47l | OrG_L_6_6 | DMN |  |
| 53 | A4hf | PrG_L_6_1 | SMN | 54 | A4hf | PrG_L_6_1 | SMN |  |
| 55 | A6cdl | PrG_L_6_2 | DAN | 56 | A6cdl | PrG_L_6_2 | DAN |  |
| 57 | A4ul | PrG_L_6_3 | SMN | 58 | A4ul | PrG_L_6_3 | SMN |  |
| 59 | A4t | PrG_L_6_4 | SMN | 60 | A4t | PrG_L_6_4 | SMN |  |
| 61 | A4tl | PrG_L_6_5 | VAN | 62 | A4tl | PrG_L_6_5 | VAN |  |
| 63 | A6cvl | PrG_L_6_6 | DAN | 64 | A6cvl | PrG_L_6_6 | DAN |  |
| 65 | A1/2/3ll | PCL_L_2_1 | VAN | 66 | A1/2/3ll | PCL_L_2_1 | VAN |  |
| 67 | A4ll | PCL_L_2_2 | SMN | 68 | A4ll | PCL_L_2_2 | SMN |  |
| 69 | A38m | STG_L_6_1 | LIM | 70 | A38m | STG_L_6_1 | LIM |  |
| 71 | A41/42 | STG_L_6_2 | SMN | 72 | A41/42 | STG_L_6_2 | SMN |  |
| 73 | TE1.0/TE1.2 | STG_L_6_3 | SMN | 74 | TE1.0/TE1.2 | STG_L_6_3 | SMN |  |
| 75 | A22c | STG_L_6_4 | SMN | 76 | A22c | STG_L_6_4 | SMN |  |
| 77 | A38l | STG_L_6_5 | LIM | 78 | A38l | STG_L_6_5 | LIM |  |
| 79 | A22r | STG_L_6_6 | DMN | 80 | A22r | STG_L_6_6 | DMN |  |
| 81 | A21c | MTG_L_4_1 | DMN | 82 | A21c | MTG_L_4_1 | DMN |  |
| 83 | A21r | MTG_L_4_2 | DMN | 84 | A21r | MTG_L_4_2 | DMN |  |
| 85 | A37dl | MTG_L_4_3 | DAN | 86 | A37dl | MTG_L_4_3 | DAN |  |
| 87 | aSTS | MTG_L_4_4 | DMN | 88 | aSTS | MTG_L_4_4 | DMN |  |
| 89 | A20iv | ITG_L_7_1 | LIM | 90 | A20iv | ITG_L_7_1 | LIM |  |
| 91 | A37elv | ITG_L_7_2 | DAN | 92 | A37elv | ITG_L_7_2 | DAN |  |
| 93 | A20r | ITG_L_7_3 | LIM | 94 | A20r | ITG_L_7_3 | LIM |  |
| 95 | A20il | ITG_L_7_4 | DMN | 96 | A20il | ITG_L_7_4 | DMN |  |
| 97 | A37vl | ITG_L_7_5 | DAN | 98 | A37vl | ITG_L_7_5 | DAN |  |
| 99 | A20cl | ITG_L_7_6 | FPN | 100 | A20cl | ITG_L_7_6 | FPN |  |
| 101 | A20cv | ITG_L_7_7 | LIM | 102 | A20cv | ITG_L_7_7 | LIM |  |
| 103 | A20rv | FuG_L_3_1 | LIM | 104 | A20rv | FuG_L_3_1 | LIM |  |
| 105 | A37mv | FuG_L_3_2 | VN | 106 | A37mv | FuG_L_3_2 | VN |  |
| 107 | A37lv | FuG_L_3_3 | DAN | 108 | A37lv | FuG_L_3_3 | DAN |  |
| 109 | A35/36r | PhG_L_6_1 | LIM | 110 | A35/36r | PhG_L_6_1 | LIM |  |
| 111 | A35/36c | PhG_L_6_2 | LIM | 112 | A35/36c | PhG_L_6_2 | LIM |  |
| 113 | TL | PhG_L_6_3 | VN | 114 | TL | PhG_L_6_3 | VN |  |
| 115 | A28/34 | PhG_L_6_4 | LIM | 116 | A28/34 | PhG_L_6_4 | LIM |  |
| 117 | TI | PhG_L_6_5 | LIM | 118 | TI | PhG_L_6_5 | LIM |  |
| 119 | TH | PhG_L_6_6 | VN | 120 | TH | PhG_L_6_6 | VN |  |
| 121 | rpSTS | pSTS_L_2_1 | DMN | 122 | rpSTS | pSTS_L_2_1 | DMN |  |
| 123 | cpSTS | pSTS_L_2_2 | VAN | 124 | cpSTS | pSTS_L_2_2 | VAN |  |
| 125 | A7r | SPL_L_5_1 | DAN | 126 | A7r | SPL_L_5_1 | DAN |  |
| 127 | A7c | SPL_L_5_2 | DAN | 128 | A7c | SPL_L_5_2 | DAN |  |
| 129 | A5l | SPL_L_5_3 | DAN | 130 | A5l | SPL_L_5_3 | DAN |  |
| 131 | A7pc | SPL_L_5_4 | SMN | 132 | A7pc | SPL_L_5_4 | SMN |  |
| 133 | A7ip | SPL_L_5_5 | DAN | 134 | A7ip | SPL_L_5_5 | DAN |  |
| 135 | A39c | IPL_L_6_1 | VN | 136 | A39c | IPL_L_6_1 | VN |  |
| 137 | A39rd | IPL_L_6_2 | FPN | 138 | A39rd | IPL_L_6_2 | FPN |  |
| 139 | A40rd | IPL_L_6_3 | DAN | 140 | A40rd | IPL_L_6_3 | DAN |  |
| 141 | A40c | IPL_L_6_4 | DMN | 142 | A40c | IPL_L_6_4 | DMN |  |
| 143 | A39rv | IPL_L_6_5 | DAN | 144 | A39rv | IPL_L_6_5 | DAN |  |
| 145 | A40rv | IPL_L_6_6 | SMN | 146 | A40rv | IPL_L_6_6 | SMN |  |
| 147 | A7m | PCun_L_4_1 | FPN | 148 | A7m | PCun_L_4_1 | FPN |  |
| 149 | A5m | PCun_L_4_2 | SMN | 150 | A5m | PCun_L_4_2 | SMN |  |
| 151 | dmPOS | PCun_L_4_3 | VN | 152 | dmPOS | PCun_L_4_3 | VN |  |
| 153 | A31 | PCun_L_4_4 | DMN | 154 | A31 | PCun_L_4_4 | DMN |  |
| 155 | A1/2/3ulhf | PoG_L_4_1 | SMN | 156 | A1/2/3ulhf | PoG_L_4_1 | SMN |  |
| 157 | A1/2/3tonIa | PoG_L_4_2 | SMN | 158 | A1/2/3tonIa | PoG_L_4_2 | SMN |  |
| 159 | A2 | PoG_L_4_3 | DAN | 160 | A2 | PoG_L_4_3 | DAN |  |
| 161 | A1/2/3tru | PoG_L_4_4 | SMN | 162 | A1/2/3tru | PoG_L_4_4 | SMN |  |
| 163 | G | INS_L_6_1 | SMN | 164 | G | INS_L_6_1 | SMN |  |
| 165 | vIa | INS_L_6_2 | SCN | 166 | vIa | INS_L_6_2 | SCN |  |
| 167 | dIa | INS_L_6_3 | VAN | 168 | dIa | INS_L_6_3 | VAN |  |
| 169 | vId/vIg | INS_L_6_4 | VAN | 170 | vId/vIg | INS_L_6_4 | VAN |  |
| 171 | dIg | INS_L_6_5 | SMN | 172 | dIg | INS_L_6_5 | SMN |  |
| 173 | dId | INS_L_6_6 | VAN | 174 | dId | INS_L_6_6 | VAN |  |
| 175 | A23d | CG_L_7_1 | DMN | 176 | A23d | CG_L_7_1 | DMN |  |
| 177 | A24rv | CG_L_7_2 | SCN | 178 | A24rv | CG_L_7_2 | SCN |  |
| 179 | A32p | CG_L_7_3 | DMN | 180 | A32p | CG_L_7_3 | DMN |  |
| 181 | A23v | CG_L_7_4 | DMN | 182 | A23v | CG_L_7_4 | DMN |  |
| 183 | A24cd | CG_L_7_5 | VAN | 184 | A24cd | CG_L_7_5 | VAN |  |
| 185 | A23c | CG_L_7_6 | VAN | 186 | A23c | CG_L_7_6 | VAN |  |
| 187 | A32sg | CG_L_7_7 | DMN | 188 | A32sg | CG_L_7_7 | DMN |  |
| 189 | cLinG | MVOcC_L_5_1 | VN | 190 | cLinG | MVOcC_L_5_1 | VN |  |
| 191 | rCunG | MVOcC_L_5_2 | VN | 192 | rCunG | MVOcC_L_5_2 | VN |  |
| 193 | cCunG | MVOcC_L_5_3 | VN | 194 | cCunG | MVOcC_L_5_3 | VN |  |
| 195 | rLinG | MVOcC_L_5_4 | VN | 196 | rLinG | MVOcC_L_5_4 | VN |  |
| 197 | vmPOS | MVOcC_L_5_5 | VN | 198 | vmPOS | MVOcC_L_5_5 | VN |  |
| 199 | mOccG | LOcC_L_4_1 | VN | 200 | mOccG | LOcC_L_4_1 | VN |  |
| 201 | V5/MT+ | LOcC_L_4_2 | DAN | 202 | V5/MT+ | LOcC_L_4_2 | DAN |  |
| 203 | OPC | LOcC_L_4_3 | VN | 204 | OPC | LOcC_L_4_3 | VN |  |
| 205 | iOccG | LOcC_L_4_4 | VN | 206 | iOccG | LOcC_L_4_4 | VN |  |
| 207 | msOccG | LOcC_L_2_1 | VN | 208 | msOccG | LOcC_L_2_1 | VN |  |
| 209 | lsOccG | LOcC_L_2_2 | VN | 210 | lsOccG | LOcC_L_2_2 | VN |  |
| 211 | mAmyg | Amyg_L_2_1 | SCN | 212 | mAmyg | Amyg_L_2_1 | SCN |  |
| 213 | lAmyg | Amyg_L_2_2 | SCN | 214 | lAmyg | Amyg_L_2_2 | SCN |  |
| 215 | rHipp | Hipp_L_2_1 | SCN | 216 | rHipp | Hipp_L_2_1 | SCN |  |
| 217 | cHipp | Hipp_L_2_2 | SCN | 218 | cHipp | Hipp_L_2_2 | SCN |  |
| 219 | vCa | BG_L_6_1 | SCN | 220 | vCa | BG_L_6_1 | SCN |  |
| 221 | GP | BG_L_6_2 | SCN | 222 | GP | BG_L_6_2 | SCN |  |
| 223 | NAC | BG_L_6_3 | SCN | 224 | NAC | BG_L_6_3 | SCN |  |
| 225 | vmPu | BG_L_6_4 | SCN | 226 | vmPu | BG_L_6_4 | SCN |  |
| 227 | dCa | BG_L_6_5 | SCN | 228 | dCa | BG_L_6_5 | SCN |  |
| 229 | dlPu | BG_L_6_6 | SCN | 230 | dlPu | BG_L_6_6 | SCN |  |
| 231 | mPFtha | Tha_L_8_1 | SCN | 232 | mPFtha | Tha_L_8_1 | SCN |  |
| 233 | mPMtha | Tha_L_8_2 | SCN | 234 | mPMtha | Tha_L_8_2 | SCN |  |
| 235 | Stha | Tha_L_8_3 | SCN | 236 | Stha | Tha_L_8_3 | SCN |  |
| 237 | rTtha | Tha_L_8_4 | SCN | 238 | rTtha | Tha_L_8_4 | SCN |  |
| 239 | PPtha | Tha_L_8_5 | SCN | 240 | PPtha | Tha_L_8_5 | SCN |  |
| 241 | Otha | Tha_L_8_6 | SCN | 242 | Otha | Tha_L_8_6 | SCN |  |
| 243 | cTtha | Tha_L_8_7 | SCN | 244 | cTtha | Tha_L_8_7 | SCN |  |
| 245 | lPFtha | Tha_L_8_8 | SCN | 246 | lPFtha | Tha_L_8_8 | SCN |  |

Note. VN, visual network; SMN, somatomotor network; DAN, dorsal attention network; VAN, ventral attention network; LIM, limbic network; FPN, frontoparietal network; DMN, default mode network; SCN, subcortical network

# References

Alexander-Bloch, A. F., Shou, H., Liu, S., Satterthwaite, T. D., Glahn, D. C., Shinohara, R. T., … Raznahan, A. (2018). On testing for spatial correspondence between maps of human brain structure and function. *NeuroImage*, *178*, 540–551. https://doi.org/10.1016/j.neuroimage.2018.05.070

Baller, E. B., Valcarcel, A. M., Adebimpe, A., Alexander-Bloch, A., Cui, Z., Gur, R. C., … Satterthwaite, T. D. (2022). Developmental coupling of cerebral blood flow and fMRI fluctuations in youth. *Cell Reports*, *38*(13). https://doi.org/10.1016/j.celrep.2022.110576

Simpson, G. L. (2018). Modelling Palaeoecological Time Series Using Generalised Additive Models. *Frontiers in Ecology and Evolution*, *6*. https://doi.org/10.3389/fevo.2018.00149

Váša, F., Seidlitz, J., Romero-Garcia, R., Whitaker, K. J., Rosenthal, G., Vértes, P. E., … Bullmore, E. T. (2018). Adolescent Tuning of Association Cortex in Human Structural Brain Networks. *Cerebral Cortex*, *28*(1), 281–294. https://doi.org/10.1093/cercor/bhx249

Wood, S. N. (2017). *Generalized additive models: An introduction with R*. chapman and hall/CRC.

Yesavage, J. A., Brink, T. L., Rose, T. L., Lum, O., Huang, V., Adey, M., & Leirer, V. O. (1982). Development and validation of a geriatric depression screening scale: A preliminary report. *Journal of Psychiatric Research*, *17*(1), 37–49. https://doi.org/10.1016/0022-3956(82)90033-4
